# The heterogeneous nutritional status trajectory and its predictors in elderly patients undergoing lung cancer surgery: a prospective cohort study

**DOI:** 10.3389/fnut.2026.1747612

**Published:** 2026-03-17

**Authors:** Shuo Shi, Jiali Yao, Chengming Fu, Xin Liu, Meiling Wang, Yue Jiao, Ling Yu

**Affiliations:** Department of Thoracic Surgery, Liaoning Cancer Hospital & Insititute, Shenyang, China

**Keywords:** elderly, latent class growth model, lung cancer, nutritional status, trajectory

## Abstract

**Objectives:**

The assessment and management of nutritional status are particularly important for reducing adverse outcomes in elderly patients undergoing lung cancer surgery. This study aimed to identify the heterogeneous nutritional status trajectory of elderly patients undergoing lung cancer surgery and analyze its predictors.

**Methods:**

A prospective cohort study was conducted in a tertiary cancer hospital in mainland China. The nutritional status of the participants was evaluated assessed on the day of admission, the day before discharge, and on the 1st, 3rd, and 6th month postoperatively. Data were analyzed using the latent class growth model, generalized linear model and logistic regression.

**Results:**

A total of 474 eligible elderly patients completed all follow-ups. Three distinct nutritional status trajectories were identified: “Severe Malnutrition-Rapid Improvement Group,” “Moderate Malnutrition-Rapid Improvement Group,” and “Persistent Low Malnutrition Risk Group.” The “Severe Malnutrition-Rapid Improvement Group” and “Moderate Malnutrition-Rapid Improvement Group” had persistent severe and moderate malnutrition, respectively, from the day before discharge to 1 month postoperatively, and both constituted populations in urgent need of clinical intervention; additionally, their quality of life across multiple domains was significantly lower than that of the “Persistent Low Malnutrition Risk Group” at 6 months postoperatively (*p* < 0.05). Therefore, these two groups were combined as the heterogeneous nutritional status trajectory. Age, TNM stage, preoperative BMI, social support level, and depression score were independent predictive factors for the heterogeneous nutritional status trajectory (*p* < 0.05).

**Conclusion:**

The nutritional status trajectories of elderly patients undergoing lung cancer surgery are highly heterogeneous and affect postoperative quality of life. Early identification and management of high-risk factors can reduce the risk of malnutrition, improve quality of life, and decrease the occurrence of adverse outcomes.

## 1 Introduction

Globally, lung cancer has the highest incidence and mortality rates among all malignant tumors, severely affecting people’s health and life (1). Due to the increasing adult population, the incidence and mortality rates of lung cancer in China are higher than the global average and are rising annually (2), leading to an increasingly severe burden of lung cancer in China (3). Statistics show that the median age at diagnosis for lung cancer is 70 years (3). As one of the fastest aging countries in the world, the number of elderly lung cancer patients in China has increased significantly (4). With the improvement of public health awareness and the development of medical imaging technology, an increasing number of patients are being diagnosed with early-stage lung cancer (5). Currently, the preferred treatment for early-stage lung cancer remains radical surgical resection (5). Surgery, as an exogenous traumatic event, can place the body in a state of stress, characterized by increased catabolism and decreased synthesis of nutrients, resulting in a significant negative nitrogen balance (6); it can also lead to the secretion of large amounts of inflammatory cytokines, which can impair immune function (7). Additionally, factors such as preoperative fasting, intraoperative blood loss, postoperative nausea and vomiting, and other discomforts that suppress appetite make patients undergoing lung cancer surgery more prone to malnutrition (8).

Malnutrition, also known as undernutrition, refers to a state of deficiency in energy or nutrients due to inadequate intake or impaired utilization (9). Studies indicate that the incidence of malnutrition in patients undergoing lung cancer surgery ranges from 24 to 55.4% (9, 10). In patients undergoing lung cancer surgery, the presence of malnutrition or nutritional risk can decrease tolerance to surgery, increase postoperative complications, prolong hospitalization, reduce quality of life, and increase mortality (11, 12). Elderly patients with lung cancer are more susceptible to malnutrition due to organ functional decline, increased comorbidities, and weakened adaptability to environmental changes (13). Malnutrition in the elderly is closely associated with frailty, sarcopenia, depression, and other adverse clinical outcomes, which further increase their hospital stays, healthcare costs, and mortality rates; it not only severely impacts their physical health and quality of life, but also places a heavy burden on families and society (14–16). Existing studies have demonstrated that early nutritional assessment and intervention for elderly patients undergoing lung cancer surgery can significantly reduce their postoperative complications and improve their physical function, quality of life, and long-term prognosis (17). Therefore, early prediction and identification of malnutrition in elderly patients undergoing lung cancer surgery, followed by timely intervention to prevent its occurrence, are crucial to reducing adverse outcomes. Furthermore, a multidisciplinary collaborative model serves as a vital approach to optimizing perioperative nutritional management in elderly patients with lung cancer. It enables the integration of multidisciplinary resources to implement individualized interventions, thereby further improving patients’ rehabilitation quality and clinical prognosis (17).

Most existing studies on the nutritional status of elderly patients undergoing lung cancer surgery were cross-sectional, focused on the current status of nutritional status at a specific time point during hospitalization and its influencing factors (18, 19), the relationship between different nutritional statuses and prognosis (12), and the effectiveness of related interventions (20, 21). However, there was a scarcity of research on the nutritional status of elderly lung cancer patients undergoing surgery during their home recovery period after discharge. Moreover, the development of nutritional status is continuous, dynamic, and heterogeneous (22, 23), in that malnutrition or nutritional risk may resolve spontaneously after discharge in some elderly patients with lung cancer, yet persist for an extended period or even deteriorate progressively in others. There is a lack of longitudinal studies exploring the changes in nutritional status in elderly patients undergoing lung cancer surgery, and the heterogeneity in the development of nutritional status has been overlooked.

Latent Class Growth Model (LCGM) is a data analysis method for identifying trends in longitudinal data (24). LCGM assumes that there are multiple latent growth trajectories within a population, each representing a subgroup with different growth patterns, allowing for heterogeneity in the study population (24). It can classify the study population into different subgroups based on categorical latent variables and describe the development trends of different subgroups and individual differences based on continuous latent variables (25, 26). LCGM has been applied in numerous studies to identify heterogeneity and developmental directions among individuals, providing a basis for the implementation of precise nursing interventions (26–28).

Currently, a variety of tools are available for the assessment of nutritional status, among which the commonly used ones include the Mini Nutritional Assessment (MNA), Patient-Generated Subjective Global Assessment (PG-SGA), Nutritional Risk Screening 2002 (NRS 2002), Malnutrition Universal Screening Tool (MUST), and Global Leadership Initiative on Malnutrition (GLIM) (17). Among these tools, the PG-SGA is a nutritional assessment instrument specifically designed for cancer patients (29). It is easy to operate, non-invasive, and requires no instrumental examination (29), allowing medical staff to complete the assessment quickly and making it particularly suitable for the longitudinal monitoring of nutritional status.

Therefore, this study intends to conduct a prospective cohort study using the PG-SGA as the main assessment tool and adopting the LCGM for data analysis. We aim to investigate the nutritional status trajectories of elderly lung cancer patients within 6 months postoperatively, identify trajectories associated with decreased quality of life on the 6th month postoperatively, merge them into the heterogeneous nutritional status trajectory and analyze its predictors. This study can provide references for the early identification of high-risk patients with malnutrition, the development of precise nutritional intervention strategies for elderly patients undergoing lung cancer surgery, the reduction of malnutrition incidence, and the improvement of quality of life.

## 2 Materials and methods

### 2.1 Study design and setting

This study was designed as a prospective cohort study conducted from October 2023 to August 2024 in the Thoracic Surgery Department of a specialized cancer hospital in Liaoning Province, China. This department was designated as a National Key Clinical Specialty. Its current nutritional management practices complied with guideline requirements. Specifically, nutritional assessments and diagnoses were conducted upon patient admission, weekly during hospitalization, post-surgery, or upon significant changes in clinical status. Patients diagnosed with moderate or severe malnutrition were reported to physicians, who then initiated intervention by the nutrition department for nutritional therapy.

This study informed prospective participants about the research objectives, methods, significance, and confidentiality of survey content, obtaining informed consent from patients. Sociodemographic data, disease characteristics, and laboratory indicators were directly retrieved from the hospital medical record system. Two nutrition specialist nurses and two nurses with master’s degrees in the research team, who had received systematic and standardized training, collected repeated measurement data of nutritional status on the day of admission (T0), the day before discharge (T1), and on the 1st (T2), 3rd (T3), and 6th (T4) month postoperatively. During hospitalization, researchers administered questionnaires on-site, reviewed completion status upon collection, and promptly addressed any omissions. Post-discharge, researchers collected data via telephone and WeChat (a Chinese mobile application with voice, text, and video call functions). Researchers adhered to the same standards as those applied in the on-site investigations, conducted item-by-item inquiries in strict accordance with the scale content, used standardized scripts during all follow-up interactions, and provided no additional medical or nursing advice to patients. Patients were considered lost to follow-up if they failed to respond to both telephone and WeChat contact attempts across three separate days.

This study was approved by the hospital’s ethics committee (approval number: KY20230901), and all participants provided informed consent. The verification was conducted by the Chinese Clinical Trial Registry (ChiCTR2400089535).

### 2.2 Participants

This study consecutively selected elderly lung cancer patients undergoing surgical treatment as participants. The inclusion criteria were as follows: patients with primary lung cancer diagnosed by preoperative or postoperative pathology (5); patients undergoing thoracoscopic surgery for the first time; patients aged ≥60 years; patients with basic understanding and language expression abilities. The exclusion criteria were: patients with consciousness disorders or mental abnormalities; patients with other serious life-threatening diseases who cannot cooperate.

This study adopted the LCGM for trajectory fitting. Given that the Bayesian information criterion should be the primary indicator, the sample size should be ≥200 (30). Considering a dropout rate of 20%, a minimum of 240 participants would be required.

### 2.3 Measurement tools

#### 2.3.1 General demographic questionnaire

Based on the literature review, this questionnaire was designed by the research team, including sociodemographic, disease characteristics, and laboratory indicators information. ① Sociodemographic information: age, gender, marital status, number of children, education level, per capita monthly family income, place of residence, living arrangements, smoking history, and alcohol consumption history. ② Disease characteristics: comorbidities, preoperative adjuvant therapy, surgical extent, pathological type, Tumor Node Metastasis (TNM) stage, postoperative nausea/ vomiting and length of hospital stay. ③ Nutritional indicators: Body Mass Index (BMI), prealbumin, total protein, albumin, hemoglobin, neutrophil and lymphocyte counts at the day of admission and the 1st day postoperatively.

#### 2.3.2 Patient-Generated Subjective Global Assessment (PG-SGA)

The PG-SGA was developed by American scholar Ottery in 1995 (29). The scale consists of two parts: the first part is a patient self-assessment form, including weight, eating status, symptoms, activity, and physical function; the second part is a medical staff assessment form, including disease-related diagnoses, stress status, and physical examination. The total score is the sum of the scores from both parts, with a score≥4 indicating malnutrition. Higher scores indicate more severe malnutrition (31). The American Society for Parenteral and Enteral Nutrition recommends the PG-SGA as the preferred tool for assessing the nutritional status of cancer patients (32). Therefore, this study used this scale to assess the nutritional status of elderly patients undergoing lung cancer surgery on the day of admission, the day before discharge, and on the 1st, 3rd, and 6th month postoperatively. In this study, the Cronbach’s alpha coefficients of the scale from T0 to T4 were 0.714, 0.688, 0.730, 0.728, and 0.721, respectively.

#### 2.3.3 Social Support Rating Scale (SSRS)

Developed by Chinese scholar (33), the SSRS includes three dimensions: objective support, subjective support, and utilization of social support, with a total of 10 items. The total score ranges from 12 to 66, with scores ≤22 indicating low social support, 23–44 indicating moderate social support, and 45–66 indicating high social support. This scale has high reliability and validity, with an internal consistency coefficient of 0.890–0.940 and a test–retest reliability of 0.926 (34). In this study, this scale was applied to assess the social support of elderly patients undergoing lung cancer surgery on the day of admission, its Cronbach’s alpha coefficient was 0.731.

#### 2.3.4 15-Item Geriatric Depression Scale (GDS-15)

The GDS-15 was created by scholar Fountoulakis by simplifying the original Geriatric Depression Scale based on the characteristics of the elderly (35). The scale consists of 15 items, with total scores ranging from 0 to 15. Higher scores indicate more severe depressive symptoms, with scores ≥8 indicating the presence of depressive symptoms (36). In this study, this scale was applied to assess the depressive situation of elderly patients undergoing lung cancer surgery on the day of admission, its Cronbach’s alpha coefficient was 0.787.

#### 2.3.5 European Organization for Research and Treatment of Cancer Quality of Life Questionnaire 30-Item Core Instrument (EORTC QLQ-C30)

The EORTC QLQ-C30 is a quality-of-life questionnaire developed by the European Organization for Research and Treatment of Cancer (37). The scale consists of 30 items across 15 domains, including one overall health status, five functional domains (physical, role, cognitive, emotional, and social function), three symptom domains (fatigue, pain, nausea and vomiting) and six single items (dyspnea, loss of appetite, sleep disturbances, constipation, diarrhea, and financial difficulties). The overall health status is scored using a 7-point Likert scale, while the remaining items use a 4-point Likert scale. Higher scores in functional domains and overall health status indicate better quality of life, while higher scores in symptom domains and single items indicate worse quality of life (37). In this study, this scale was applied to assess the quality-of-life of elderly patients undergoing lung cancer surgery on the 6th month postoperatively, its Cronbach’s alpha coefficient was 0.873.

### 2.4 Statistical analysis

Data analysis was performed using SPSS 26.0 and Mplus 8.3 software, with a two-sided test and a significance level of *p* < 0.05. Normally distributed continuous data were described using means and standard deviations, with group comparisons using t-tests; non-normally distributed continuous data were described using medians and interquartile ranges, with group comparisons using non-parametric rank-sum tests; categorical data were described using frequencies and percentages, with group comparisons using chi-square tests or Fisher’s exact tests.

The PG-SGA scores at each time point in this study were found to be non-normally distributed according to the Kolmogorov–Smirnov test. Therefore, the Friedman test was used to compare the overall differences in PG-SGA scores at different time points. The LCGM was used to explore different trajectory classes of PG-SGA scores. Three models, linear, quadratic and freely estimated, were used to predict the growth trends. The Akaike information criterion (AIC), Bayesian information criterion (BIC), sample-size-adjusted Bayesian information criterion (aBIC), entropy, bootstrapped likelihood ratio test (BLRT), and Lo–Mendell–Rubin (LMR) were used to evaluate the fit of the trajectories. Lower AIC, BIC and aBIC values indicate better model fit; entropy values range from 0 to 1, with values closer to 1 indicating more precise classification; LMR and BLRT with *p* < 0.05, indicate that k classes are better than k-1 classes (38).

Generalized linear models were used to examine the relationship between different trajectories and quality of life on the 6th month postoperatively, identifying heterogeneous nutritional status trajectory. To minimize the influence of potential confounding variables, the models were adjusted for clinically relevant sociodemographic characteristics (age, gender, marital status, number of children, education level, per capita monthly family income, place of residence, living arrangements, smoking history, and alcohol consumption history), disease characteristics (comorbidities, preoperative adjuvant therapy, surgical extent, pathological type, TNM stage, postoperative nausea/ vomiting and length of hospital stay), and preoperative nutritional indicators (BMI, prealbumin, total protein, albumin, hemoglobin, neutrophil and lymphocyte counts).

To address potential multicollinearity issues in the general data, Variance Inflation Factors (VIF) were used for diagnosis, with VIF > 10 indicating severe multicollinearity (39). Variables were screened or adjusted accordingly. Finally, multivariate logistic regression analysis was used to explore the influencing factors of heterogeneous nutritional status trajectory.

## 3 Results

### 3.1 General demographic characteristics of participants

On the day of admission (T0), a total of 527 questionnaires were distributed. On the 1st day postoperatively, 27 patients with benign pathology were excluded. Follow-up was conducted for the remaining 500 elderly patients undergoing lung cancer surgery. After 6 months of follow-up, 474 patients completed all follow-ups. A total of 26 patients were lost to follow-up (3 patients died, 4 patients voluntarily withdrew from the study, and 18 patients failed to respond to both telephone and WeChat), with a dropout rate of 5.20%, of which 4 patients were lost to follow-up at the 1st month postoperatively (T2), 7 patients at the 3rd month postoperatively (T3), and 15 patients at the 6th postoperatively (T4).

Among the 474 elderly patients with lung cancer in this study, 46.4% were male and 53.6% were female. The age range was 60–82 years. 45.1% of patients had a smoking history, and 39.9% had a history of alcohol consumption. The majority of patients (88.2%) had adenocarcinoma as their pathological type, and most patients (81.2%) were in TNM stage I. The average postoperative hospital stay for patients was 6.17 days. Detailed information is shown in Table 1.

**Table 1:** Univariate analysis of predictive factors of heterogeneous nutritional status trajectories.

Variables	Total (*n* = 474)	Heterogeneous nutritional status trajectory (*n* = 258)	Persistent low malnutrition risk group (*n* = 216)	*χ^2^/t* values	*p*-values
Age, *n*(%)				10.582	**0.001**
<65	126(26.6)	53(20.5)	73(33.8)		
≥65	348(73.4)	205(79.5)	143(66.2)		
Gender, *n*(%)				0.480	0.488
Male	220(46.4)	116(45.0)	104(48.1)		
Female	254(53.6)	142(55.0)	112(51.9)		
Marital status, *n*(%)				0.327	0.567
Unmarried/Divorced/Widowed	38(8.0)	19(7.4)	19(8.8)		
Married	436(92.0)	239(92.6)	197(91.2)		
Number of children, *n*(%)				0.362	0.547
1	254(53.6)	135(52.3)	119(55.1)		
≥2	220(46.4)	123(47.7)	97(44.9)		
Education level, *n*(%)				0.915	0.633
Junior and below	304(64.1)	168(65.1)	136(63.0)		
Senior	97(20.5)	54(20.9)	43(19.9)		
College and above	73(15.4)	36(14.0)	37(17.1)		
Per capita monthly family income, *n*(%)				2.179	0.536
<1,000	91(19.2)	50(19.4)	41(19.0)		
1,000–2,999	129(27.2)	76(29.5)	53(24.5)		
3,000–4,999	112(23.6)	61(23.6)	51(23.6)		
≥5,000	142(30.0)	71(27.5)	71(32.9)		
Residence, *n*(%)				5.920	**0.015**
Urban	315(66.5)	159(61.6)	156(72.2)		
Rural	159(33.5)	99(38.4)	60(27.8)		
Living arrangements, *n*(%)				0.765	0.682
Living alone	47(9.9)	28(10.9)	19(8.8)		
Living with spouse	374(73.2)	203(78.7)	171(79.2)		
Others	53(11.2)	27(10.5)	26(12.0)		
Smoking history, *n*(%)				0.009	0.923
No	260(54.9)	141(54.7)	119(55.1)		
Yes	214(45.1)	117(45.3)	97(44.9)		
Alcohol consumption history, *n*(%)				0.347	0.556
No	285(60.1)	152(58.9)	133(61.6)		
Yes	189(39.9)	106(41.1)	83(38.4)		
Comorbidities, *n*(%)				0.926	0.336
No	219(46.2)	114(44.2)	105(48.6)		
Yes	255(53.8)	144(55.8)	111(51.4)		
Preoperative adjuvant therapy, *n*(%)				0.337	0.562
No	456(96.2)	247(95.7)	209(96.8)		
Yes	18(3.8)	11(4.3)	7(3.2)		
Surgical extent, *n*(%)				1.687	0.430
Lung segmental resection	87(18.4)	46(17.8)	41(19.0)		
Lobectomy	282(59.5)	149(57.8)	133(61.6)		
Lung wedge resection	105(22.1)	63(24.4)	42(19.4)		
Pathological type, *n*(%)				0.022	0.882
Adenocarcinoma	418(88.2)	227(88.0)	191(88.4)		
Others	56(11.8)	31(12.0)	25(11.6)		
TNM stage, *n*(%)				7.449	**0.006**
I	385(81.2)	198(76.7)	187(86.6)		
≥II	89(18.8)	60(23.3)	29(13.4)		
Postoperative nausea and vomiting, *n*(%)				2.777	0.096
No	349(73.6)	182(70.5)	167(77.3)		
Yes	125(26.4)	76(29.5)	49(22.7)		
Social support, *n*(%)				21.916	**<0.001**
Medium	382(80.6)	228(88.4)	154(71.3)		
High	92(19.4)	30(11.6)	62(28.7)		
Depression scores, *n*(%)				6.128	**0.025**
<8	464(97.9)	249(96.5)	215(99.5)		
≥8	10(2.1)	9(3.5)	1(0.5)		
**Length of hospital stay,** $\left(\bar{x}\pm s\right)$	6.17 ± 2.35	6.23 ± 2.45	6.10 ± 2.23	0.623	0.534
Preoperative					
BMI (kg/m^2^), $\left(\bar{x}\pm s\right)$	24.22 ± 3.26	23.89 ± 3.37	24.62 ± 3.07	2.078	**0.015**
Prealbumin (mg/L), $\left(\bar{x}\pm s\right)$	255.09 ± 50.75	250.74 ± 54.00	260 ± 46.16	−2.074	**0.039**
Total albumin (g/L), $\left(\bar{x}\pm s\right)$	71.16 ± 4.82	70.55 ± 4.58	71.67 ± 4.96	−2.525	**0.012**
Albumin (g/L), $\left(\bar{x}\pm s\right)$	44.48 ± 3.64	43.59 ± 3.29	45.23 ± 3.76	−4.981	**<0.001**
Hemoglobin (g/L), $\left(\bar{x}\pm s\right)$	141.11 ± 14.56	141.40 ± 14.99	140.75 ± 14.06	0.483	0.630
Neutrophil count (10^9/L), $\left(\bar{x}\pm s\right)$	3.98 ± 3.69	3.93 ± 3.83	4.04 ± 3.52	−0.328	0.743
Lymphocyte count (10^9/L), $\left(\bar{x}\pm s\right)$	1.78 ± 1.77	1.66 ± 0.53	1.92 ± 0.55	−1.601	0.110
Postoperative					
Total albumin (g/L), $\left(\bar{x}\pm s\right)$	62.23 ± 5.80	61.89 ± 6.11	62.63 ± 5.40	−1.375	0.170
Albumin (g/L), $\left(\bar{x}\pm s\right)$	37.22 ± 3.87	37.20 ± 4.11	37.25 ± 3.57	−0.135	0.893
Hemoglobin (g/L), $\left(\bar{x}\pm s\right)$	127.79 ± 15.92	126.79 ± 16.7	128.98 ± 15.56	−1.497	0.135
Neutrophil count (10^9/L), $\left(\bar{x}\pm s\right)$	8.72 ± 4.66	8.03 ± 3.10	9.54 ± 5.92	−3.557	**<0.001**
Lymphocyte count (10^9/L), $\left(\bar{x}\pm s\right)$	1.24 ± 0.79	1.20 ± 0.49	1.28 ± 1.03	−1.176	0.240

Bold values represent statistical significance.

### 3.2 Nutritional status at different time points during the perioperative period in elderly lung cancer patients

In this study, the PG-SGA scores of elderly patients with lung cancer on the day of admission (T0), the day before discharge (T1), and on the 1st (T2), 3rd (T3), and 6th (T4) month postoperatively were 2(2,3), 7(5,9), 6(4,9), 3(2,4), and 2(2,3), the differences at each time point were statistically significant (*χ^2^* = 1508.288, *p* < 0.001). Nutritional risks in elderly patients undergoing lung cancer surgery showed a trend of first increasing and then decreasing, reaching a maximum at the day before discharge and remaining at a high level between the day before discharge and the 1st month postoperatively.

On the day of admission, 19.41% of elderly lung cancer patients exhibited malnutrition; on the day before discharge, 90.71% exhibited malnutrition; 1 month post-surgery, 84.18% exhibited malnutrition; 3 months post-surgery, 32.7% exhibited malnutrition; 6 months post-surgery, 4.43% exhibited malnutrition.

### 3.3 Trajectories of nutritional status in elderly patients undergoing lung cancer surgery

The PG-SGA scores at five-time points were fitted to explore the potential classes of nutritional status trajectories in elderly patients undergoing lung cancer surgery. The results showed that the freely estimated three-class model was the optimal model, with an entropy value of 0.831, and both LMR and BLRT reached significant levels (*p* < 0.05). The AIC (8808.926), BIC (8875.505), and aBIC (8824.723) values were all relatively low. Detailed model fit indices are shown in Table 2.

**Table 2:** The fitting indicators of each model.

Class	AIC	BIC	aBIC	Entropy	LMR	BLRT	Class probability
Linear							
1C	10702.779	10731.908	10709.691	–	–	–	1
2C	10525.818	10567.430	10535.692	0.960	0.025	<0.001	0.898/0.102
3C	10462.716	10516.811	10475.551	0.786	0.201	<0.001	0.096/0.728/0.176
4C	10069.211	10135.790	10085.009	1.000	0.114	<0.001	0.278/0.156/0.011/0.555
5C	10075.211	10154.274	10093.971	1.000	0.243	<0.001	0.278/0.011/0.156/0.555/0.000
Quadratic							
1C	10522.399	10551.528	10529.311	–	–	–	1
2C	10301.627	10343.239	10311.500	0.752	0.034	<0.001	0.241/0.759
3C	10216.822	10270.917	10229.658	0.851	<0.001	<0.001	0.309/0.680/0.011
4C	9627.616	9694.195	9643.413	1.000	0.737	<0.001	0.156/0.044/0.555/0.245
5C	9598.158	9677.221	9616.918	0.986	0.770	<0.001	0.044/0.156/0.028/0.245/0.527
Free estimation							
1C	9605.746	9647.358	9615.620	–	–	–	1
2C	8978.097	9032.193	8990.933	0.857	<0.001	<0.001	0.438/0.562
**3C**	**8808.926**	**8875.505**	**8824.723**	**0.831**	**0.004**	**<0.001**	**0.130/0.452/0.418**
4C	8673.364	8752.427	8692.124	0.865	0.355	<0.001	0.324/0.086/0.321/0.269
5C	8617.278	8708.825	8639.000	0.885	0.007	<0.001	0.315/0.012/0.268/0.318/0.087

1C = the one-class model; 2C = the two-class model; 3C = the three-class model; 4C = the four-class model; 5C = the five-class model; AIC = Akaike Information Criterion; BIC=Bayesian Information Criterion; aBIC = sample-corrected Bayesian Information Criterion; BLRT = Bootstrap Likelihood Ratio Test; LMR = Lo–Mendell–Rubin. Bold values represent statistical significance.

**Figure 1:**
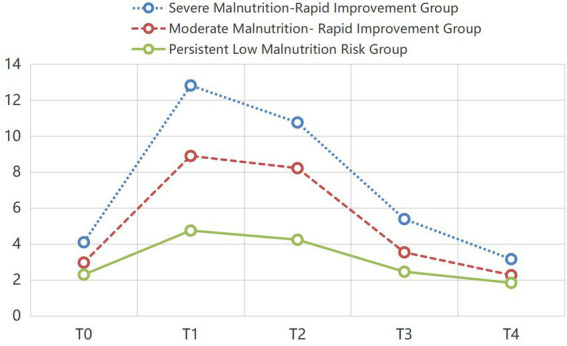
Trajectories of nutritional status in elderly patients undergoing lung cancer surgery. T0 = the day of admission; T1 = the day before discharge; T2 = the 1st month postoperatively; T3 = the 3rd month postoperatively; T4 = the 6th month postoperatively.

A trajectory graph of the three potential classes was drawn with PG-SGA scores on the *y*-axis and postoperative follow-up time on the *x*-axis (Figure 1). According to the PG-SGA scoring method (40), 0–1 for no malnutrition, 2–3 for possible malnutrition, 4–8 for moderate malnutrition, and ≥9 for severe malnutrition, as well as the recovery speed of each group, the three trajectory classes were named as follows: ① Class 1: This group had the highest PG-SGA scores, with a mean peak value of 12.84 on the day before discharge, and the fastest recovery rate (*slope* = 8.189, *p* < 0.001), thus named “Severe Malnutrition-Rapid Improvement Group”, including 62 patients, accounting for 13.08%; ② Class 2: This group had higher PG-SGA scores, with a mean peak value of 8.90 on the day before discharge, and a relatively fast recovery rate (*slope* = 6.043, *p* < 0.001), thus named “Moderate Malnutrition-Rapid Improvement Group”, including 196 patients, accounting for 41.35%; ③ Class 3: This group had the lowest PG-SGA scores, with a mean peak value of only 4.75 on the day before discharge, and consistently low nutritional risk at all time points (*slope* = 2.546, *p* < 0.001), thus named “Persistent Low Malnutrition Risk Group”, including 216 patients, accounting for 45.57%.

### 3.4 Comparison of quality of life at the 6th month postoperatively among different nutritional status trajectories

We used generalized linear models to analyze the relationship between nutritional status trajectories and quality of life. The results showed that at the 6th month postoperatively, compared with the “Persistent Low Malnutrition Risk Group,” the “Severe Malnutrition-Rapid Improvement Group” had significantly lower scores in overall health (*p* < 0.001), physical functioning (*p* = 0.001), cognitive functioning (*p* = 0.004), and significantly higher severity in fatigue (*p* < 0.001), pain (*p* < 0.001), dyspnea (*p* < 0.001), loss of appetite (*p* < 0.001); the “Moderate Malnutrition-Rapid Improvement Group” had significantly lower overall health scores (*p* < 0.001) and significantly higher severity in fatigue (*p* < 0.001), pain (*p* = 0.002), and dyspnea (*p* < 0.001). More detailed information is shown in Table 3.

**Table 3:** Heterogeneity nutritional status trajectory analysis based on generalized linear model.

Domain	Class 1 vs. Class 3^a^		Class 2 vs. Class 3^a^	
	*β*(95%CI)	*p*	*β*(95%CI)	*p*
**Overall health status**	−13.351(−16.651, −10.052)	**<0.001**	−7.299(−9.558, −5.040)	**<0.001**
Functional domains				
Physical	−5.109(−8.049, −2.170)	**0.001**	−0.534(−2.546, 1.479)	0.270
Role	0.132(−2.537, 2.801)	0.923	0.565(−1.262, 2.393)	0.544
Emotional	0.587(−1.815, 2.990)	0.632	0.428(−1.217, 2.073)	0.610
Cognitive	−5.473(−9.175, −1.771)	**0.004**	−1.075(−4.818, 2.669)	0.574
Social	2.526(−1.531, 6.583)	0.222	0.521(−2.257, 3.299)	0.713
Symptom domains				
Fatigue	12.088(8.766, 15.410)	**<0.001**	4.095(1.820, 6.370)	**<0.001**
Nausea and vomiting	−0.463(−1.580, 0.660)	0.417	−0.038(−0.803, 0.727)	0.923
Pain	15.162(12.825, 17.498)	**<0.001**	2.477(0.878, 4.077)	**0.002**
Single items				
Dyspnea	6.570(3.050, 13.383)	**<0.001**	4.856(2.446, 7.266)	**<0.001**
Loss of appetite	21.729(17.878, 25.580)	**<0.001**	2.353(−0.284, 4.989)	0.080
Sleep disturbances	1.474(−1.945, 4.892)	0.398	0.195(−2.146, 2.536)	0.870
Constipation	−0.772(−2.259, 0.716)	0.309	−0.261(−1.280, 0.757)	0.615
Diarrhea	0.304(−1.180, 1.787)	0.688	−0.261(−1.277, 0.754)	0.614
Financial difficulties	−1.707(−5.277, 1.862)	0.348	0.054(−2.390, 2.497)	0.966

Class 1 = Severe Malnutrition-Rapid Improvement Group; Class 2 = Moderate Malnutrition-Rapid Improvement Group; Class 3 = Persistent Low Malnutrition Risk Group; ^a^Use Class 3 as reference. Bold values represent statistical significance.

The “Severe Malnutrition-Rapid Improvement Group” and the “Moderate Malnutrition-Rapid Improvement Group” experienced persistent severe and moderate malnutrition, respectively, from the day before discharge to 1 month postoperatively. They exhibited similar magnitude and rate of improvement, and both represent populations in urgent need of clinical intervention. Furthermore, their quality of life in multiple domains was significantly lower than that of the “Persistent Low Malnutrition Risk Group” at the 6th month postoperatively. Therefore, the “Severe Malnutrition-Rapid Improvement Group” and the “Moderate Malnutrition-Rapid Improvement Group” were combined into the “Heterogeneous nutritional status trajectory” in this study, including 258 patients, accounting for 54.43%.

### 3.5 Predictors of heterogeneous nutritional status trajectory

On the Univariate analysis results showed that 10 variables were significantly associated with heterogeneous nutritional status trajectory in elderly patients undergoing lung cancer surgery (*p* < 0.05): age, place of residence, TNM stage, social support, depression, preoperative BMI, preoperative prealbumin, preoperative total protein, preoperative albumin, and neutrophil count postoperatively (see Table 1 for details). Binary logistic regression analysis showed that age, TNM stage, preoperative BMI, social support, and depression were independent predictive factors for heterogeneous nutritional status trajectory in elderly patients undergoing lung cancer surgery (*p* < 0.05) (see Table 4 for details).

**Table 4:** Multifactorial analysis of predictive factors of heterogeneous nutritional status trajectories.

Variables	*β*	SE	Wald	*p*	OR	95%CI
Age						
<65	−0.662	0.226	7.569	**0.006**	0.537	(0.345, 0.836)
≥65	Reference					
Residence						
Urban	−0.316	0.217	2.126	0.145	0.729	(0.476, 1.115)
Rural	Reference					
TNM stage						
I	−0.609	0.266	5.236	**0.022**	0.544	(0.323, 0.916)
≥II	Reference					
Social support						
Medium	0.879	0.260	11.401	**0.001**	2.409	(1.446, 4.016)
High	Reference					
**Depression scores**	0.255	0.064	15.742	**<0.001**	1.291	(1.138, 1.465)
**Preoperative BMI**	−0.064	0.031	4.122	**0.042**	0.938	(0.883, 0.998)
**Preoperative prealbumin**	0.001	0.002	0.091	0.762	1.001	(0.996, 1.005)
**Preoperative total albumin**	−0.008	0.026	0.087	0.768	0.992	(0.944, 1.044)
**Preoperative albumin**	−0.008	0.036	0.052	0.820	0.992	(0.924, 1.065)
**Postoperative neutrophil count**	0.048	0.029	2.613	0.106	1.049	(0.990, 1.111)
**Constant**	2.460	2.072	1.409	0.235	–	–

Bold values represent statistical significance.

## 4 Discussion

Due to the invasiveness of thoracoscopic surgery, elderly lung cancer patients are likely to face unavoidable nutritional risks postoperatively. This longitudinal study comprehensively explored the nutritional status of 474 elderly lung cancer patients from hospital admission to 6 months after surgery at different time points, highlighting the heterogeneity in postoperative nutritional trajectories. To our knowledge, this was the first longitudinal trajectory study to report the nutritional status of elderly lung cancer patients during the perioperative period. These findings not only reveal the dynamic and heterogeneous nature of nutritional status in elderly patients undergoing lung cancer surgery, but also provide a theoretical basis for formulating more effective and precise nutritional intervention strategies, ultimately contributing to improving patients’ nutritional status, quality of life, and clinical outcomes.

The results of this study showed that the nutritional risk in elderly patients with lung cancer peaks on the day before discharge and remained high from the day before discharge to the 1st month postoperatively. On the day of admission, only 19.4% of elderly patients undergoing lung cancer surgery had nutritional risk or malnutrition, which is similar to previous studies (12, 18). However, the proportion of patients with nutritional risk or malnutrition significantly increased to 90.7% on the day before discharge and 84.2% on the 1st month postoperatively. This may be due to the significant trauma of lung cancer surgery, which causes various discomforts in the early postoperative period, such as pain, dyspnea, nausea and vomiting, and fatigue, leading to reduced appetite, decreased food intake, and significantly reduced activity levels (41). Additionally, preoperative fasting and postoperative early diet restrictions (only consuming liquid and semi-liquid foods) fail to meet the patients’ nutritional needs. Subsequently, the incidence of nutritional risk or malnutrition gradually decreased, dropping to 32.7% on the 3rd month postoperatively and 4.4% on the 6th month postoperatively. This indicates that the nutritional status of elderly patients undergoing lung cancer surgery is dynamic, with a high incidence of malnutrition or nutritional risk, and its improvement requires a relatively long time. These results highlight the necessity of continuous monitoring of nutritional status among elderly patients undergoing lung cancer surgery within 1 month after surgery (the high-risk period for nutritional risk or malnutrition). Future studies may conduct high-frequency monitoring of nutritional status among elderly lung cancer patients in the early postoperative period to accurately identify the peak time point of nutritional risk, and prolong the postoperative follow-up duration to fully capture the dynamic changes in postoperative nutritional status.

In this study, three distinct nutritional status trajectories were identified in elderly patients undergoing lung cancer surgery, showing significant heterogeneity. Previous studies have mostly adopted a cross-sectional perspective, providing nutritional interventions immediately when patients exhibit nutritional risk or malnutrition at a specific time point (21, 42). However, the nutritional status trajectories in this study reveal that some patients, specifically those in the “Persistent Low Malnutrition Risk Group,” may have nutritional risk preoperatively or in the early postoperative period, but their nutritional status can improve autonomously through surgery or short-term postoperative adjustment without requiring special clinical intervention.

This study also identified the “moderate malnutrition–rapid improvement” group and the “severe malnutrition–rapid improvement” group, both of which exhibited poor preoperative nutritional status. This highlights the value for healthcare providers to conduct early nutritional assessment and implement timely interventions before surgery. The “Moderate Malnutrition-Rapid Improvement Group” experienced moderate malnutrition within 1 month postoperatively, with significant improvement in nutritional status by 3 months postoperatively. These patients require additional attention from healthcare providers, with appropriate interventions in the early postoperative period to help them through this critical period of poor nutritional status. The “Severe Malnutrition-Rapid Improvement Group” experienced severe malnutrition within 1 month postoperatively, with persistent malnutrition at the 3rd month postoperatively and remaining at risk of malnutrition at the 6th month postoperatively. The continued presence of malnutrition not only reduces the effectiveness of treatment but also increases the risk of readmission and healthcare costs, further affecting overall prognosis and quality of life (43). Therefore, the nutritional issues of these patients need further attention, and healthcare providers should offer more intensive and personalized nutritional interventions to improve long-term outcomes.

Postoperative quality of life is increasingly used to assess whether surgery is truly successful (44). The results of this study show that nutritional status trajectories significantly impact the postoperative quality of life of elderly patients with lung cancer, especially the “Moderate Malnutrition-Rapid Improvement Group” and “Severe Malnutrition-Rapid Improvement Group”. These findings emphasize the potential negative impact of malnutrition on quality of life, while indicating that comprehensive perioperative nutritional assessment and personalized nutritional support represent key steps in improving prognosis (42). The results of the generalized linear model show that compared with the “Persistent Low Malnutrition Risk Group,” the “Severe Malnutrition-Rapid Improvement Group” had a significantly lower quality of life in terms of overall health, physical functioning, and cognitive functioning, and significantly higher severity in fatigue, pain, dyspnea, and loss of appetite. This indicates that persistent malnutrition or high risk of malnutrition may lead to greater physiological and cognitive stress, as well as more severe symptoms of fatigue, pain, dyspnea, and loss of appetite, thereby reducing postoperative quality of life (45–47). Similarly, the “Moderate Malnutrition-Rapid Improvement Group” also had significantly lower quality of life in multiple domains at the 6th month postoperatively, suggesting that if moderate to severe malnutrition is not identified and managed in a timely manner in the early postoperative period, it may significantly affect the patients’ quality of life, and this negative impact may not have completely disappeared even at the 6th month postoperatively (46). Therefore, this study combined the “Severe Malnutrition-Rapid Improvement Group” and the “Moderate Malnutrition-Rapid Improvement Group” into the “heterogeneous nutritional status trajectory,” further emphasizing the heterogeneous impact of different nutritional status trajectory classes on patient quality of life. These patients represent the high-risk population truly in need of intervention. It is essential to identify these patients early, closely monitor changes in their nutritional status, and provide effective interventions in a timely manner to improve their nutritional status, accelerate the recovery of physiological and cognitive functions, and enhance their quality of life.

This study identified age, TNM stage, preoperative BMI, social support level, and depression score as independent predictive factors for the heterogeneous nutritional status trajectory, providing a scientific basis for reducing the occurrence of the heterogeneous nutritional status trajectory in clinical practice.

Firstly, compared with patients aged 60–64 years, elderly patients undergoing lung cancer surgery aged ≥65 years were more likely to develop a heterogeneous nutritional status trajectory. The reason may be that with increasing age, elderly patients are more prone to physiological functional decline, such as loose and falling teeth, decreased appetite, and weakened digestive and absorptive functions, leading to inadequate nutrient intake and impaired utilization, thereby increasing the incidence of malnutrition (48). This suggests that it is necessary for clinical practice to establish a multidisciplinary team to conduct comprehensive evaluations of diet, anthropometry, physical function, and social activities in elderly patients with lung cancer, especially before surgery and before discharge, and to provide them with professional and comprehensive nutritional management to improve their nutritional status and quality of life (17). Internationally, an age of ≥65 years is commonly used as the threshold for defining elderly individuals. However, our study adopted ≥60 years as the cutoff for elderly patients, mainly based on the statutory definition in the Law of the People’s Republic of China on the Protection of the Rights and Interests of the Elderly that “elderly citizens refer to those aged 60 years and above” (49), and also to more accurately reflect the nutritional status of elderly Chinese patients with lung cancer. To ensure the comparability of our findings with similar international studies, we further stratified elderly patients into two subgroups: 60–64 years and ≥65 years, and explored the differences in nutritional status between these two groups. Future studies may continue to investigate the differences in nutritional status among elderly individuals of different age groups to facilitate better stratified management.

Secondly, compared with patients in TNM stage I, those in stage II and above had a higher probability of developing a heterogeneous nutritional status trajectory. The reason may be that elderly patients undergoing lung cancer surgery in TNM stage II and above usually require multimodal treatments, such as neoadjuvant therapy before surgery or chemotherapy, targeted therapy, or immunotherapy after surgery (5). These treatments often cause various discomforts, such as nausea, vomiting, decreased appetite, cough and fatigue (50), which further limit patients’ nutrient intake and increase the risk of malnutrition. This suggests that clinical practice should conduct follow-up visits for elderly patients undergoing lung cancer surgery in TNM stage II and above after discharge and provide different nutritional support strategies at different treatment stages, using digital health technologies to regularly and dynamically assess and monitor patients’ nutritional status and adherence to nutritional interventions to reduce the occurrence of malnutrition.

Thirdly, elderly patients undergoing lung cancer surgery who had higher preoperative depression scores were more likely to develop a heterogeneous malnutrition trajectory, consistent with previous studies (48). The reason may be that depression can cause sympathetic nerve excitation, leading to weakened gastrointestinal motility and worsening symptoms of indigestion (51). Additionally, elderly patients undergoing lung cancer surgery often have significant psychological burdens due to concerns about disease prognosis, potential postoperative discomforts or complications, and medical expenses, which can lead to negative emotions, lethargy and decreased appetite, thereby affecting their nutritional status. This suggests that clinical practice should pay attention to the psychological health status of elderly patients undergoing lung cancer surgery, and it is crucial to identify and manage depressive symptoms early. Regular psychological assessments, counseling, psychotherapy, or pharmacotherapy can be conducted perioperatively to alleviate patients’ depressive symptoms.

Fourthly, elderly patients undergoing lung cancer surgery who had higher preoperative BMI were less likely to develop a heterogeneous malnutrition trajectory. Previous studies have also shown that being overweight and mildly obese have a protective effect on the body’s nutritional status (19, 52). BMI can directly reflect the body’s energy reserve levels, and patients with low BMI often experience lipid metabolism disorders due to insufficient fat reserves and surgical stress, leading to a lack of anti-inflammatory mediators, exacerbating inflammatory responses and immune suppression (11). However, this result should be interpreted with caution, as sarcopenic obesity is highly prevalent among cancer patients (53). BMI fails to reflect body composition, and muscle mass has far greater clinical significance than fat mass in the assessment of nutritional reserves. This indicates that in clinical practice, it is essential not only to enhance the dynamic monitoring of perioperative BMI but also to conduct dynamic measurement of muscle mass via specialized approaches such as body composition analysis, which is of great value for assessing patients’ nutritional status and determining their prognosis.

Finally, elderly patients undergoing lung cancer surgery who had higher preoperative social support levels were less likely to develop a heterogeneous malnutrition trajectory, indicating that enhancing patients’ social support level may be an effective intervention strategy. Previous studies have shown that social support can improve patients’ health-promoting behaviors, enhance self-efficacy and treatment adherence, and help them face the disease with a positive attitude (54, 55). Therefore, effective intervention measures should be taken to increase patients’ social support levels, making it easier for patients to obtain regular dietary assistance and timely nutritional intervention resources to improve their nutritional status. In this study, the SSRS was used to assess social support. Although this scale is a widely adopted and well-validated instrument among the Chinese population with sound psychometric properties, and its dimensional structure is consistent with Chinese cultural norms, its international application is limited, which poses potential challenges to the cross-cultural comparability of the present study’s findings. This is mainly because social support is a culture-bound construct. In the Chinese cultural context, social support is usually reciprocal, collective, and family-centered, with an emphasis on “objective support” and “utilization of support” (33). In contrast, Western countries tend to prioritize individual-centered support, focusing on “subjectively perceived support” and “instrumental support” (56). Therefore, the findings of this study regarding the association between social support and nutritional status should be interpreted with caution when generalized to non-Chinese populations, and further research is warranted to explore their association.

Although this study provides important insights, it also has some limitations. First, although PG-SGA is a well-validated and widely accepted tool in oncology for nutritional assessment and risk stratification, it showed suboptimal reliability at T1 in the present study. In addition, it lacks the formal malnutrition diagnosis based on GLIM. Future studies may adopt the GLIM criteria for nutritional status assessment when both the patient’s condition and clinical environment permit, so as to improve the standardization and comparability of malnutrition diagnosis. Second, although data collectors strictly adhered to quality control principles throughout the follow-up period, telephone follow-up may inevitably exert subtle influences on participants (such as enhancing their treatment compliance and their attention to their own health status). This may act as a confounder in the present study and affect the observed nutritional trajectories and quality of life outcomes. For future research, it is suggested to utilize mobile health technologies to automatically send out questionnaires to patients at each follow-up time point, so as to minimize the impacts arising from human factors. Third, during the postoperative recovery phase, biochemical indicators such as albumin, prealbumin, and total protein levels can be affected by systemic inflammatory responses and may not reliably reflect the true nutritional status when used alone. Therefore, it is recommended that future studies combine inflammatory markers for multidimensional nutritional assessment to enhance the accuracy of nutritional evaluation. Fourth, among elderly patients with tumors, malnutrition and sarcopenia frequently coexist and interact in a bidirectional causal relationship. Sarcopenia may already present in the early stage, even when no significant changes occur in patients’ dietary intake or body weight. However, due to constraints related to clinical environment and manpower, the present study did not evaluate the skeletal muscle mass of the patients. Future studies are recommended to carry out specialized screening for sarcopenia in elderly patients with tumors, which can identify occult nutritional and functional impairments at the preclinical stage, provide a basis for the timely implementation of precise nutritional or exercise interventions, optimize the perioperative physiological and functional status of elderly patients with tumors, and ultimately improve their long-term prognosis. Fifth, this is a single-center study conducted in a specialized cancer hospital. Although the nutritional management implemented in the study is in line with clinical guidelines, the nutritional trajectories identified are inevitably influenced by the hospital-specific model of perioperative diagnosis, treatment and nutritional management, which may limit the generalizability of the study results to other regions. Future multicenter studies are warranted to enroll medical institutions at different levels for comparative analyses, so as to formulate more targeted and hierarchical nutritional management strategies for this population. Finally, the identified nutritional trajectories represent statistical groupings derived from the LCGM and should therefore be interpreted as probabilistic patterns rather than strictly deterministic clinical categories. Given that trajectory modeling depends on model assumptions and fit indices, a cautious interpretation is warranted when translating these classes into clinical decision-making pathways.

## 5 Conclusion

This study revealed the dynamicity and heterogeneity of perioperative nutritional status among elderly patients undergoing lung cancer surgery, and identified three distinct nutritional status trajectories. Patients with heterogeneous nutritional trajectories exhibited poorer quality of life at 6 months after surgery, which highlights the necessity of dynamic stratified nutritional assessment during the perioperative period. In addition, this study found that age, TNM stage, preoperative BMI, social support level, and depression score were independent predictive factors of heterogeneous nutritional status trajectories. This suggests that perioperative nutritional management for elderly patients with lung cancer should integrate multidisciplinary resources, combine the stratified characteristics of nutritional trajectories, and formulate individualized and dynamically adjustable nutritional intervention programs through long-term postoperative follow-up, so as to improve patients’ nutritional status and quality of life, and reduce the occurrence of adverse outcomes. This study provides theoretical and practical evidence for the implementation of precision nutritional management during the perioperative period in elderly patients with lung cancer, and has important clinical value and promotional significance for promoting the standardized and precise development of nutritional management in elderly cancer patients.

## Data Availability

The original contributions presented in the study are included in the article/supplementary material, further inquiries can be directed to the corresponding author.
